# Resolving versus non-resolving sphingolipid dynamics during macrophage activation: a time-resolved metabolic analysis

**DOI:** 10.1016/j.jlr.2025.100899

**Published:** 2025-09-15

**Authors:** Nathan F. Chiappa, Nidhi Lal, Edward A. Botchwey

**Affiliations:** 1 Wallace H. Coulter Department of Biomedical Engineering, Georgia Institute of Technology and Emory University School of Medicine, Atlanta, GA, USA; 2 Parker H. Petit Institute for Bioengineering and Bioscience, Georgia Institute of Technology, Atlanta, GA, USA; 3 Department of Biomedical Engineering, Emory University School of Medicine, Atlanta, GA, USA

**Keywords:** sphingolipids, sphingosine phosphate, lipidomics, inflammation, ceramides

## Abstract

Sphingolipids are increasingly recognized as critical regulators of inflammation and cell fate decisions, with metabolites such as ceramide and sphingosine 1-phosphate exerting contrasting effects on cell survival and proliferation. In macrophages, this balance is especially important, given their central role in host defense, pathogenesis and wound healing. Here, we present a time-resolved model of sphingolipid metabolism in RAW 264.7 macrophages stimulated with KdO_2_–Lipid A. By integrating measured metabolite concentrations with dynamic flux estimation and established enzyme kinetics, we systematically map dynamic changes in the sphingolipid network during inflammation. Our results reveal a three-phase pattern of sphingolipid remodeling that correlates with distinct functional states of the cell. Moreover, metabolites can be classified into “resolving” or “non-resolving” lipids based on whether they return to basal levels or remain dysregulated through the later phases of the inflammatory response. This partitioning suggests that targeted modulation of specific metabolic nodes may influence the resolution of inflammation. Importantly, our computational approach can assist in the rational design of experimental studies by pinpointing putative drug targets with maximal impact on sphingolipid homeostasis. Such targeted interventions may prevent the pathological amplification of inflammatory signals without globally suppressing essential sphingolipid functions. These findings highlight the utility of an integrative systems-level analysis for elucidating sphingolipid dynamics in macrophages and underscore its potential to guide therapeutic strategies against conditions involving dysregulated inflammation.

Macrophages are key orchestrators of immune responses, functioning at the crossroads of innate and adaptive immunity (1). They are essential for mounting potent inflammatory responses—producing cytokines, phagocytosing pathogens, and modulating other immune cells—while also contributing to wound healing and tissue homeostasis. However, excessive or dysregulated macrophage activation can drive the pathogenesis of numerous conditions, including atherosclerosis (2), tissue fibrosis (3, 4, 5), and cancer (6). Increasingly, research points to lipid metabolism as a decisive factor in these activation states (7, 8). Lipids not only supply membrane components for proliferating or migrating cells but also regulate signal transduction pathways that govern macrophage polarization and function. These diverse functional states require precise metabolic coordination, particularly in lipid metabolism, which provides both energy and signaling molecules.

Among lipids, sphingolipids have garnered particular attention for their potent bioactive properties in controlling cell fate and inflammatory processes (6, 9). Two key metabolites, ceramide and sphingosine-1-phosphate (S1P), often exhibit opposing functions. Ceramide typically favors pro-apoptotic outcomes and cell cycle arrest, whereas S1P supports pro-survival and proliferative outcomes (10, 11, 12). These metabolites are interconnected within a complex metabolic network, where ceramide is created de-novo as well as by the interconversion of complex sphingolipids, and can be converted into sphingosine and subsequently phosphorylated to form S1P (7, 9). This "sphingolipid rheostat" maintains a delicate balance between pro-survival and pro-apoptotic signals (13), and dysregulation at any point can lead to pathological outcomes like chronic inflammation and tissue damage (3). Indeed, aberrant sphingolipid metabolism has been implicated in a range of inflammatory pathologies, from metabolic disorders to fibrosis and neurodegenerative conditions (14, 15).

To capture this complexity and identify critical nodes within this network, integrative systems-level approaches are essential (16, 17). Key enzymes in this network include sphingomyelinases that generate ceramide, ceramidases that convert ceramide to sphingosine, and sphingosine kinases that produce S1P (18). By merging experimental measurements of metabolite levels with computational modeling, these methods clarify how ceramide, S1P, and their interconversions influence macrophage polarization (e.g., M1 versus M2 phenotypes), cytokine production, and antigen-presentation capacities (15, 19). For example, inhibiting acid ceramidase disrupts MHC class II antigen presentation (19), while reinforcing the Spns2/S1P axis prevents hyperinflammation and late-stage immunosuppression (15). Understanding the timing of these metabolic shifts is crucial for elucidating both protective and pathogenic immune responses (7, 20).

Despite significant progress in mapping sphingolipid pathways, critical gaps remain in our understanding of their temporal dynamics and flux regulation. Most studies measure lipid levels at only a single or limited number of time points (8, 9), providing a static snapshot rather than capturing the dynamic trajectory of how cells transition through different activation states. Furthermore, while large-scale lipidomic databases (e.g., LIPID MAPS) have facilitated the identification and quantification of numerous sphingolipid species (20), few efforts have integrated these data into flux or enzyme-activity analyses to reveal which enzymatic steps truly govern the observed changes (6, 17). Without this flux information, it remains unclear whether targeting a particular enzyme is a viable anti-inflammatory strategy or would merely induce inconsequential shifts in metabolite levels.

To address these gaps in understanding sphingolipid dynamics during inflammation, we developed an integrated experimental and computational approach combining time-resolved lipidomics with flux balance analysis. This strategy allowed us to address two fundamental questions: 1) How do changes in metabolic flux and enzyme activity drive the temporal evolution of sphingolipid concentrations during macrophage activation? and 2) Can systematic analysis of these fluxes identify optimal targets for anti-inflammatory intervention? By mapping the dynamic relationships between enzyme activities, metabolic fluxes, and lipid concentrations, our work provides a quantitative framework for understanding how macrophages coordinate sphingolipid metabolism during inflammation and reveals new opportunities for therapeutic modulation of inflammatory responses.

## Materials and methods

### Experimental design and data analysis

**Fig. 1:**
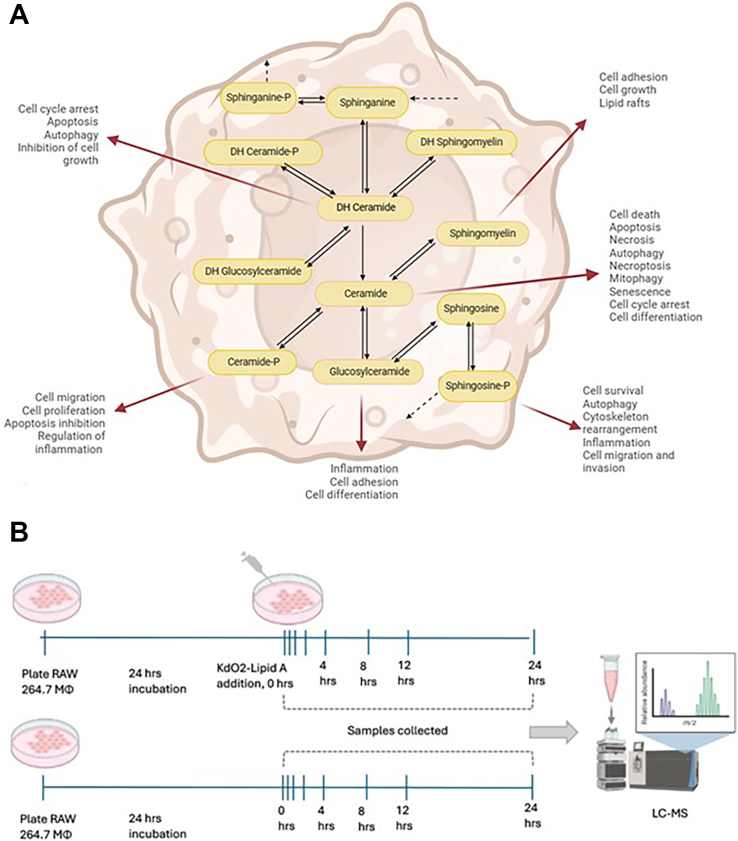
A: Macrophage sphingolipids as key players in inflammation. This schematic highlights the interconnected sphingolipid network, centered on ceramide as a central “hub” metabolite that links upstream precursors (dihydrosphingosine, dihydrosphingosine-P, dihydroceramide, and their phosphorylated or glycosylated derivatives) to downstream effectors (sphingomyelin, glucosylceramide, sphingosine, and sphingosine-P). Solid arrows denote enzymatic conversions; dashed arrows represent irreversible reactions. Each metabolite exerts distinct yet overlapping roles in regulating macrophage biology—including inflammation, apoptosis, necroptosis, autophagy, cell migration, and survival—thus underscoring the duality of sphingolipid signaling. Ceramide predominantly drives pro-apoptotic and pro-inflammatory responses, whereas sphingosine-P promotes cell survival and cytoskeletal rearrangements. By modulating cell adhesion, proliferation, and differentiation, changes in sphingolipid metabolism can profoundly influence macrophage-mediated inflammation and immune function. B: Experimental design for LIPID MAPS time-course analysis of RAW 264.7 macrophages with or without KdO_2_-Lipid A stimulation. After 24 h of initial culture, KdO_2_-Lipid A (stimulated condition) or vehicle only (unstimulated control) was added, and cells were harvested at 0, 0.5, 1, 2, 4, 8, 12, and 24 h. Collected samples were processed and analyzed by LC–MS to evaluate temporal changes in the macrophage lipidome.

Sphingolipids form an interconnected network centered on ceramide, with each metabolite influencing distinct aspects of macrophage function (Fig. 1A). To investigate temporal dynamics of sphingolipid metabolism during macrophage activation, we analyzed comprehensive lipidomic data from RAW 264.7 macrophages responding to KdO_2_-Lipid A (KLA) stimulation. Specifically, we utilized the time-course dataset from Dennis *et al.* (20, 21) (https://www.lipidmaps.org/resources/protocols/PP0000001004.pdf; https://www.lipidmaps.org/data/results/raw2647/kdo2lipidatimecourse/view_2_t.php?LIST_OF_EXPTS=17,18,22,250,251,252,253,254&expt=kla), in which RAW 264.7 macrophages cultured in 10% serum were treated with 100 ng/ml KLA. KLA is a component of lipopolysaccharide (LPS) with confirmed TLR4-mediated bioactivity comparable to LPS (21). Samples were harvested at 0, 0.5, 1, 2, 4, 8, 12, and 24 h for both stimulated and unstimulated controls, and LC–MS measurements were collected for a variety of sphingolipids (Fig. 1B). For each sphingolipid class, the concentrations of all molecular species (i.e., different acyl-chain variants) were summed at each time point to yield total “pool” concentrations. Because raw measurements in the LIPID MAPS database were provided in units different from enzyme activity values in literature, we converted them to pmol/mg protein, using the relationship that 3 μg of DNA ≈ 0.25 mg protein (17). Literature-based activity parameters were aggregated to approximate enzyme kinetics in RAW 264.7 cells (Table 1). However, since direct measurements for this specific cell line were scarce, composite enzyme activity values from other white blood cell types were used as proxies.

**Table 1:** Enzyme Activities Taken from Literature

Enzyme Name	Species	Cell/Tissue	Substrate Used	Activity (pmole/min/mg Protein)	Average Activity (pmole/min/mg Protein)	Reference
CERK	human	PMN	-	1.5	28.25	(22)
CERK	human	HL-60	d18:0/24:1 CER	55		(23)
Acid SMase	mouse	neutrophil mito		0.116666667	39.52916667	(24)
Acid SMase	mouse	neutrophil mito		0.116666667		(24)
Acid SMase	human	HL-60	16:0 or 18:0 SM	320		(25)
Acid SMase	human	HL-60	LR-C12-SM	58.33333333		(26)
Acid SMase	human	NB4	-	50		(27)
Acid SMase	human	HL-60	-	0.666666667		(28)
Neutral SMase	human	HL-60	-	1.833333333		(28)
Neutral SMase	human	HL-60	16:0 or 18:0 SM	41.33333333		(25)
Neutral SMase	human	HL-60	C6-NBD-SM	1.333333333		(29)
Acid SMase	human	Molt-4	bovine brain SM	0.1		(30)
Neutral SMase	human	Molt-4	bovine brain SM	0.15		(30)
Neutral SMase	human	Jurkat	C6-NBD-SM	0.366666667		(31)
SMS	human	HL-60	C6 NBD CER	16.66666667	3044.45	(28)
SMS	human	HL-60	C6-NBD-CER	2.916666667		(29)
SMS	human	leukemia	C6 NBD CER	1.666666667		(28)
SMS	human	Jurkat	C6 NBD CER	15200		(32)
SMS	human	Jurkat	C6 NBD CER	1		(31)
GLUCERS	human	leukemia	C6 NBD CER	8.333333333	1796.642857	(31)
GLUCERS	mouse	pbmc	C8-CER	1.333333333		(33)
GLUCERS	human	Jurkat	C6 NBD CER	12400		(32)
GLUCERS	human	HL-60	C6 NBD CER	8.5		(34)
GLUCERS	human	HL-60	C16 CER	16.66666667		(35)
GLUCERS	human	HL-60	C6 NBD CER	100		(28)
GLUCERS	human	HL-60	C6-NBD-CER	41.66666667		(29)
DES1	Rat	Rat hepatocytes	DHCER	16.66666667	16.66666667	(36)
LCS	Chinese hamster	CHOP	NBD-GluCer	3.66666666	5.58333333	(37)
LCS	Human	Human dermal fibroblast- Golgi fraction	GlcCer	7.5		(38)
Acid CDase	human	HL-60 cells	C16 CER	15.83333333		(35)
Neutral CDase	human	HL-60	C16 CER	0		(36)
Alkaline CDase	human	HL-60 cells	C16 CER	6.666666667		(36)
Acid CDase	human	lymphoid	RBM14-12	166.6666667		(39)
Acid CDase	human	lymphoid	C12 BODIPY CER	0.055		(40)
Alkaline CDase	human	Jurkat	C12 NBD PCER	9	33.03694445	(41)
GLUCDase	human	HL-60	4-MU	120000		(42)
GLUCDase	human			333.3333333	60166.66667	(43)
Sk1	human	NB4	Sph	4	19.13333333	(44)
SK1				50		(45)
SK1	human	Jurkat	NBD-SPH	2.666666667		(46)
SK1	human	KU812F	SPH	30		(44)
SK1	human	NALM-17	SPh	9		(44)
SK2	human	Nb4	Sph	900		(44)
SK2				50		(45)
SK	human	Jurkat	SPH	6		(47)
SK2	human	Jurkat	NBD-SPH	8		(46)
SK2	human	KU812F	SPh	1100		(44)
SK2	human	NALM-17	SPh	800	477.3333333	(44)
S1PPase				100	100	(45)
CERS	mouse	P388 microsomes	DHSPH, Pal-CoA	14		(48)
CERS	human	Molt-4	SPH, Pal-CoA	22.4	18.2	(49)
CERS	human	PBMC		positive		(50)
SPT	human	Lymphocyte microsome	Pal-CoA	1.125		(51)
SPT	human	Jurkat microsomes	Pal-CoA	650		(32)
SPT	human	Molt-4	Pal-CoA	5.95	219.025	(49)
S1PL	mouse	liver	S1P	111	111	(52)
LPP	human	HL-60	C6-NBD-C1P	2400	2400	(53)
GALCDase	mouse	liver		31.66666667	46.11233333	(54)
GALCDase	human	Lymphocyte	C7 GALCER	9.728333333		(55)
GALCDase	human	Lymphocyte	bovine brain GALCER	113.3333333		(56)
GALCDase	human	lymphocyte	bovine brain GALCER	49.33333333		(57)
GALCDase	human	leukocyte		26.5		(58)

### Metabolic network construction

**Fig. 2:**
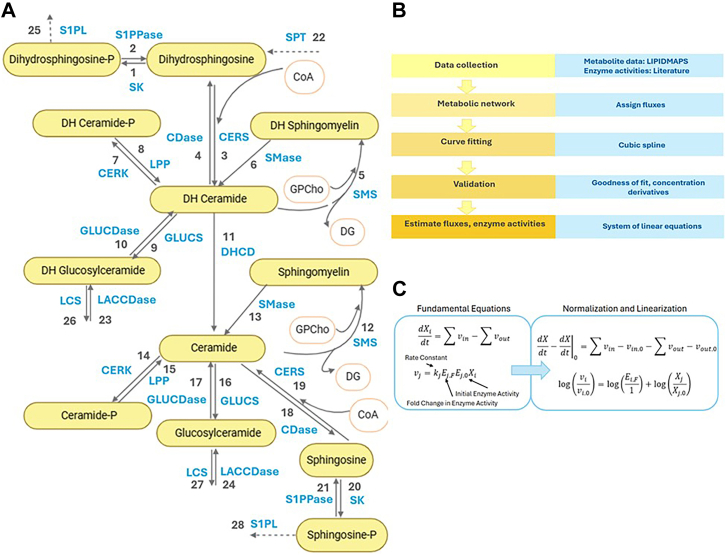
Dynamic flux estimation for the sphingolipid metabolic network. A: Metabolic network diagram showing key enzyme-mediated steps (numbered) in the sphingolipid pathway of KdO_2_-Lipid A–stimulated RAW 264.7 macrophages. Export processes for each sphingolipid are not shown on the network map. B: Algorithmic workflow for flux estimation. Metabolite concentration data (from LIPID MAPS) and literature-based enzyme activities are integrated into a time-dependent metabolic network. Fluxes are assigned, and a cubic spline–based approach is used for curve fitting. The model is validated by comparing the goodness-of-fit and time-derivatives of measured concentrations, culminating in quantitative estimates of fluxes and enzyme activities. C: Fundamental equations and key variables. Shown are the ordinary differential equations (ODEs) governing metabolite concentrations, along with linearized expressions. Rate constants, initial enzyme activities, and fold changes in enzyme activity are incorporated, enabling dynamic flux inferences from experimental data. Abbreviations: CDase, ceramidase; CERK, ceramide kinase; CERS, ceramide synthase; DHCD, dihydroceramide desaturase; GLUCDase, glucosylceramidase; GLUCS, glucosylceramide synthase; LACCDase, lactosylceramidase; LCS, lactosylceramide synthase; LPP, lipid phosphate phosphatase; S1PL, sphingosine 1-phosphate lyase; S1PPase, sphingosine 1-phosphate phosphatase; SMase, sphingomyelinase; SMS, sphingomyelin synthase; SK, sphingosine kinase; SPT, serine palmitoyltransferase. Images created with BioRender.

A sphingolipid metabolic network was constructed based on published literature, the KEGG database, and previously established knowledge of enzyme-mediated reactions. To systematically analyze the temporal evolution of sphingolipid metabolism, we constructed a detailed metabolic network incorporating all measured species and their enzymatic interconversions (Fig. 2A). The network comprises twelve sphingolipid metabolites (Table 2), each assigned a unique variable (X_i_), connected by enzyme-mediated reactions and export processes designated by specific flux numbers (v_i_). Fifteen key enzymes (Table 3) catalyze 28 distinct reactions, with 12 additional export processes (Table 4). This network structure enabled quantitative flux analysis using the computational workflow (Fig. 2B, C).

**Table 2:** Dependent Variable Identification

Sphingolipid Name	Abbreviation	Variable
Dihydro Sphingosine	DHSph	X1
Dihydro Sphingosine 1 Phosphate	DHSphP	X2
Dihydro Ceramide	DHCer	X3
Dihydro Sphingomyelin	DHSM	X4
Dihydro Ceramide 1 Phosphate	DHCerP	X5
Dihydro Glucosylceramide	DHGluCer	X6
Ceramide	Cer	X7
Sphingomyelin	SM	X8
Ceramide 1 Phosphate	CerP	X9
Glucosylceramide	GluCer	X10
Sphingosine	Sph	X11
Sphingosine 1 Phosphate	SphP	X12

**Table 3:** Independent Variable Identification

Enzyme Name	Abbreviation	Variable
Sphingosine Kinase	SK	X13
S1P Phosphatase	S1PPase	X14
Ceramide Synthase	CERS	X15
Ceramidase	CDase	X16
Sphingomyelin Synthase	SMS	X17
Sphingomyelinase	SMase	X18
Ceramide Kinase	CERK	X19
Lipid Phosphate Phosphatase	LPPase	X20
Glucosylceramide Synthase	GLUCERS	X21
Glucosylceramidase	GLUCDase	X22
Dihydroceramide Desaturase	DHCD	X23
Serine Palmitoyltransferase	SPT	X24
Lactosylceramidase	LACCDase	X25
S1P Lyase	S1PL	X26
Lactosylceramide Synthase	LACCERS	X27
Other Variables		
Acyl Coenzyme A	CoA	X28
Glycerophosphatidylcholine	GPCho	X29

**Table 4:** Flux Identification

Reaction	Catalysing Enzyme	Flux Number
DHSph → DHSphP	SK	v1
DHSphP→ DHSph	S1PPase	v2
DHSph+ CoA → DHCer	CERS	v3
DHCer→ DHSph	CDase	v4
DHCer + GPCho → DHSM + DG	SMS	v5
DHSM → DHCer	SMase	v6
DHCer → DHCerP	CERK	v7
DHCerP → DHCer	LPPase	v8
DHCer → DHGluCer	GLUCERS	v9
DHGluCer → DHCer	GLUCDase	v10
DHCer → Cer	DHCD	v11
Cer + GPCho → SM + DG	SMS	v12
SM → Cer	SMase	v13
Cer → CerP	CERK	v14
CerP → Cer	LPPase	v15
Cer → GluCer	GLUCERS	v16
GluCer → Cer	GLUCDase	v17
Cer → Sph	CDase	v18
Sph + CoA → Cer	CERS	v19
Sph → SphP	SK	v20
SphP → Sph	S1PPase	v21
→ DHSph	SPT	v22
→ DHGluCer	LACCDase	v23
→ GluCer	LACCDase	v24
DHSphP →	S1PL	v25
DHGluCer→	LACCERS	v26
GluCer →	LACCERS	v27
SphP →	S1PL	v28
DHSph →	Export	v29
DHSphP →	Export	v30
DHCer →	Export	v31
DHSM →	Export	v32
DHCerP →	Export	v33
DHGluCer→	Export	v34
Cer→	Export	v35
SM →	Export	v36
CerP →	Export	v37
GluCer→	Export	v38
Sph →	Export	v39
SphP →	Export	v40

### Mass balance and rate equations

The temporal evolution of sphingolipid concentrations can be described through mass balance equations that account for all production and degradation processes. For each metabolite X_i_, the rate of change is given by:

$$\frac{dX_{i}}{dt}=\sum v_{in,i}-\sum v_{out,i}$$

where v_in,i_ represents all fluxes entering X_i_, and v_out,i_ represents all fluxes leaving X_i_. Each flux (v_i_) is associated with an enzymatic (or export) reaction in the network. The general rate equation for a flux v_i_ was:

$$\nu_{i}=k_{i}E_{i}X_{i}=k_{i}E_{F,i}E_{0,i}X_{i}=k_{i}^{\prime }E_{0,i}X_{i}$$

where k_i_ is the rate constant, E_i_ is the effective enzyme activity at a given time, E_0,i_ is the baseline (initial) enzyme activity, E_F,i_ is the fold change of that enzyme relative to baseline, X_i_ is the relevant substrate concentration, and k’_i_ is the product of k_i_ and E_F,i_ which we refer to as the rate parameter. Any unmeasured cofactors were assumed to be in sufficient excess so that their concentration remained effectively constant, and their effects were lumped into the rate constants (k_i_).

Table 5 illustrates how ceramide (X_7_) participates in multiple reactions (e.g., conversion from dihydroceramide, sphingomyelin, glucosylceramide, and sphingosine). Hence, the ceramide balance equation is: $dX_{7}/dt=v_{11}+v_{13}+v_{15}+v_{17}+v_{19}-\left(v_{12}+v_{14}+v_{16}+v_{18}+v_{35}\right)$ and each flux v_i_ is expressed as: $v_{11}=k_{11}X_{23}X_{3}$, $v_{13}=k_{13}X_{18}X_{8}$, $v_{15}=k_{15}X_{20}X_{9}$, $v_{17}=k_{17}X_{22}X_{10}$, $v_{19}=k_{19}X_{15}X_{11}X_{28}$ $v_{12}=k_{12}X_{17}X_{7}X_{20}$, $v_{14}=k_{14}X_{19}X_{7}$, $v_{16}=k_{16}X_{21}X_{7}$, $v_{18}=k_{18}X_{16}X_{7}$, and $v_{35}=k_{35}X_{7}$. Unmeasured substrates (e.g., ATP, UDP-Glucose, or other cofactors) are included in the rate constants k_i_ as they are assumed constant. Combining the flux-balance equations for all sphingolipids yields a vector form: dX/dt=Sv where: X is the vector of sphingolipid concentrations, v is the vector of fluxes, and S is the stoichiometric matrix, specifying how each flux contributes to each sphingolipid’s production or consumption. Figure 2B illustrates our overall modeling workflow, and Figure 2C summarizes the core rate equations.

**Table 5:** Ceramide as an example of flux balance setup

Flux No.	Reaction	Enzyme
11	Dihydroceramide (X_3_) → Ceramide (X_7_)	DHCD (X_23_)
13	Sphingomyelin (X_8_) → Ceramide (X_7_)	SMase (X_18_)
15	Ceramide 1-phosphate (X_9_) → Ceramide (X_7_)	LPP (X_20_)
17	Glucosylceramide (X_10_) → Ceramide (X_7_)	GluCDase (X_22_)
19	Sphingosine (X_11_) + CoA (X_28_) → Ceramide (X_7_)	CERS (X_15_)
12	Ceramide (X_7_) + GPCho (X_29_) → SM (X_8_) + DG	SMS (X_17_)
14	Ceramide (X_7_) → Ceramide 1-phosphate (X_9_)	CERK (X_19_)
16	Ceramide (X_7_) → GluCer (X_10_)	GLUCERS (X_21_)
18	Ceramide (X_7_) → Sphingosine (X_11_)	CDase (X_16_)
35	Ceramide (X_7_) → Export	— (export)

### Spline fitting, derivatives and numerical implementation

**Supplementary Figure S1:**
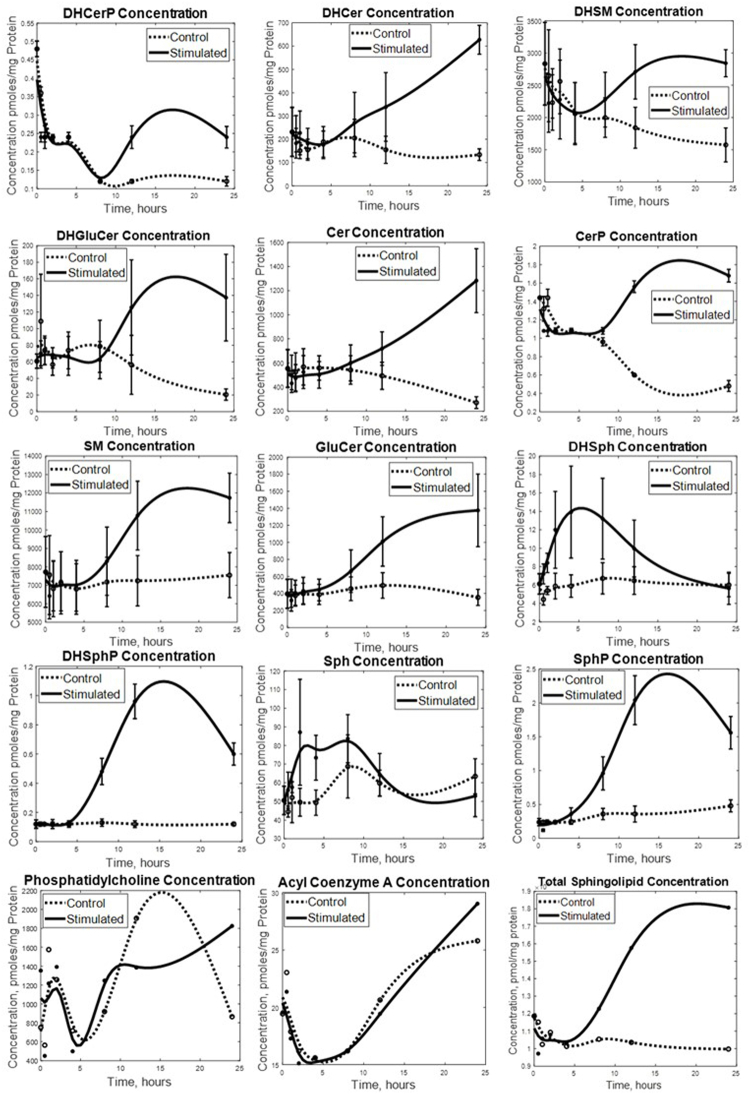
Experimental Concentration Data Were Fit to Cubic Smoothing Spline Functions. Data from control macrophages (dotted line) and stimulated macrophages (solid line) were fit to cubic smoothing spline function in MATLAB. Error bars were added based on biological replicate data in the LIPID MAPS data set.

Metabolic fluxes were estimated over the time course of the experiment using dynamic flux estimation (DFE) (59). Each sphingolipid’s measured concentration over time was fitted to a cubic smoothing spline (MATLAB’s csaps function) with a manually adjusted smoothing parameter to ensure a visually good fit (supplemental Fig. S1). We then computed time derivatives of these spline fits (using the fnder function), providing numerical values of (dX_i_/dt) at each sampled time point. These derivatives, together with the stoichiometric matrix S, yield a system of equations: dX/dt=Sv. We solved for the flux vector ν at each time point by minimizing the sum of squared errors ${\left\|dX/dt-Sv\;\right\|}^{2}$ via the MATLAB lsqlin function, subject to bounds: lb≤v≤ub. Lower bounds (lb) for each flux were set to 0 and upper bounds (ub) were set to a multiple of the corresponding enzyme activity taken from literature. The multiple was manually adjusted to ensure the sum of squared errors of the optimal solution was no greater than 10^-10^. From these optimized fluxes, we calculated rate constants and enzyme fold changes. Rate constants (k_i_) were determined from initial conditions: $k_{i}=\frac{v_{i,0}}{E_{i,0}X_{i,0}}$ while enzyme fold changes (E_F,i_) were computed throughout the time course: $E_{F,i}=\frac{v_{i}}{k_{i}E_{i,0}X_{i}}$. Predicted trajectories for each flux are shown in supplemental Fig. S2 and predicted trajectories for each enzyme activity are shown in supplemental Fig. S3.

### Confidence interval analysis

The variability present in the LIPID MAPS data set affects the uncertainty in the model predictions. In order to quantify that uncertainty, we took an approach similar to that of Chen *et al.* (16). Briefly, for each lipid/time point, we created a normal distribution with the same mean and standard error as the experimental data. We then randomly sampled 1,000 points from each normal distribution to create a set of 1,000 data sets with the same means and standard errors as the LIPID MAPS data set. We then, performed the same parameter estimation procedure on all 1,000 data sets, collected the results and calculated percentiles for each prediction. Since the LIPID MAPS concentration data are shown as mean ± standard error of the mean, which is the 68% confidence interval of the mean, we show model predictions as the mean and a band containing the middle 68% of the simulation results.

### Global pattern analysis

To identify system-wide patterns in sphingolipid metabolism, we performed principal component analysis (PCA) on three key datasets: smoothed metabolite concentrations, calculated fluxes, and inferred enzyme activities. For each analysis, time points were treated as samples, with variables centered and normalized using MATLAB's pca function. This approach revealed coordinated changes across the sphingolipid network during macrophage activation.

### Dynamic sensitivity analysis

The fully parameterized differential equation model enabled investigation of how perturbations in enzyme activities affect sphingolipid concentrations dynamically. To do this, we first define the sensitivity of lipid concentration Xi with respect to enzyme activity Ej as $s_{i,j}=\frac{\partial X_{i}}{\partial E_{j}}$. Next, we calculate the time derivative of s_i,j_ as $\frac{ds_{i,j}}{dt}=\frac{d}{dt}\left(\frac{\partial X_{i}}{\partial E_{j}}\right)=\frac{\partial }{\partial E_{j}}\left(\frac{dX_{i}}{dt}\right)$. Remembering that $\frac{dX_{i}}{dt}=\sum v_{in,i}-\sum v_{out,i}$, this becomes $\frac{ds_{i,j}}{dt}=\frac{\partial }{\partial E_{j}}\left(\sum v_{in,i}-\sum v_{out,i}\right)$. We end up with 12 × 15 = 180 differential equations for sensitivities that must be integrated together with the original 12 differential equations for lipid concentrations to generate estimates of dynamic sensitivities (60). It is often convenient to express sensitivities in relative or scaled terms as $S_{i,j}=\frac{\partial X_{i}}{\partial E_{j}}\frac{E_{j}}{X_{i}}=\frac{\partial \ln\left(X\right)}{\partial \ln\left(E\right)}$. This dimensionless sensitivity coefficient (S_i,j_) measures the relative change in metabolite concentration (X_i_) resulting from a relative change in enzyme activity (E_j_), providing insight into key regulatory points in the network (61).

### Cell culture

RAW 264.7 cells were expanded in Dulbecco’s Modified Eagle Medium (Gibco 11965118), supplemented with 10% FBS (Corning 35-010-CV) and 1% Penicillin-Streptomycin. The cultures were expanded and seeded onto appropriate well plates at a seeding density of about 105,000 cells/cm^2^ (P4-P5) and allowed to attach overnight. Cells in the treatment group were dosed with 100 ng/ml KdO2- Lipid A (Avanti 699500).

### Flux tracking

Metabolic fluxes were evaluated using a protocol modified from Nikolova-Karakashian (62). 10 uM C6- NBD SM as a tracer was added at 0 h to both control and KLA-treated groups. Media and cell (PBS suspension) samples were collected at 0, 4, 12, and 24 h after KdO2- Lipid A, where the media samples were centrifuged at 170 × g for 7 min to remove any floating cells. Media was collected from the wells and the cells were washed once with PBS and scraped to detach them from the surface. Samples were stored at −80°C and later thawed on ice. They were then sonicated, and 50 μL of each sample was aliquoted for total protein estimation using the BCA assay. 400 μL of the rest of the samples were aliquoted into glass lipid extraction tubes. C6 NBD DHCer as an internal standard, as well as 1 ml of NBD-Sphingolipid mobile phase consisting of 850 ml MeOH, 150 ml H_2_O, and 1.5 ml Phosphoric acid, were added and allowed to incubate in a 48°C water bath overnight with shaking. The samples were centrifuged at 1000xg for 10 min, and the top 1 ml of the solution was transferred to HPLC autosampler vials, without disturbing the bottom, to remove settled material. The samples were analysed using a Shimadzu HPLC with fluorescence detector (ex/em: 460/535 nm). Peak areas were converted to concentrations using a calibration curve.

Metabolic fluxes were estimated using a simplified flux balance model including only the NBD-lipid metabolites in the media and in the cells. The differences in fluxes between the KLA-stimulated cells and the control cells were calculated and compared to the model predictions using the LIPID MAPS data. NBD-lipid concentrations with curve fitting are shown in supplemental Fig. S6.

### Enzyme activity assay

Acid and neutral SMase activities were evaluated using a protocol modified from Nikolova-Karakashian (62). Media was removed from the wells and the cells were washed with PBS before scraping (in PBS) and storing at −80°C. The samples were later thawed on ice and sonicated for cell lysis. 50 μL of each sample was aliquoted for total protein estimation using the BCA assay. Based on the assay, an amount of sample containing 5 ug of protein was added into Eppendorf tubes, and 400 uL of the appropriate buffer (acid/neutral) containing 5 uM C6-NBD SM was added into each tube. The acid buffer is a 0.5 M acetate buffer containing sodium acetate and glacial acetic acid in DI water, pH 4.5, and the neutral buffer is a 10 mM Tris Buffer containing Tris HCl, Tris base, 5 mM MgCl2, and 0.2% (w/v) Triton X-100, pH 7.4. The reaction tubes were placed in a shaking water bath at 37°C for 1 h for the reaction to proceed. 1 ml of the NBD-Sphingolipid mobile phase described previously was added to each tube. The samples were transferred to lipid extraction tubes and put back in the shaking water bath for another hour. They were then centrifuged at 1000g for 10 min to pellet insoluble material. 1 ml of the supernatant was transferred to autosampler tubes and analysed by a Shimadzu HPLC with fluorescence detection (ex/em: 460/535 nm).

### Total protein estimation

Total protein was estimated using a Pierce BCA assay kit (23225). 0, 0.05, 0.1, 0.2, 0.4, 0.8, and 2 mg/ml BSA were used as assay standards. 10 μL of the media or cell homogenates were added in triplicates to a 96-well plate, and subsequently 190 μL of the BCA reagent solution containing reagents A and B in the ratio 50:1 was added. The plate was covered with aluminum foil and incubated for 30 min at 37°C and read on a plate reader (SpectraMax M2, Molecular Devices) at 562 nm.

## Results

### PCA reveals a Three-Phase Sphingolipid response

**Fig. 3:**
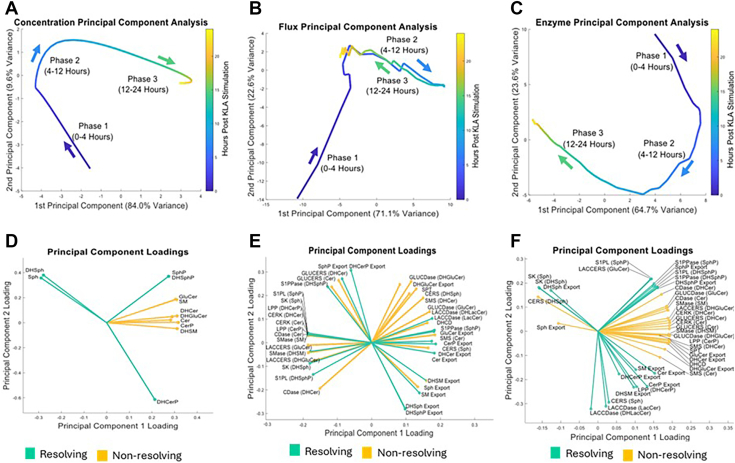
Principal component analysis (PCA) reveals a three-phase response in macrophage sphingolipid metabolism. A–C: PCA of (A) concentrations, (B) fluxes, and (C) enzyme activities each displays a similar three-phase trajectory (Phase 1: 0–4 h, Phase 2: 4–12 h, Phase 3: 12–24 h). The color scale indicates sampling time; arrows highlight transitions between phases. Despite measuring different levels (lipids, fluxes, or enzymes), all datasets shift coherently from early to intermediate to late time points, suggesting coordinated but distinct metabolic dynamics. (D–F) Loadings plots for the first two principal components show which sphingolipid species (D), fluxes (E), and enzymes (F) most strongly drive each phase of the response. Variables with large positive or negative loadings on PC1 or PC2 are key contributors to overall variance. Clustering patterns help pinpoint specific molecular processes (e.g., ceramide synthesis or sphingosine phosphorylation) that define early or late metabolic transitions. Overall, these PCA results confirm that the macrophage sphingolipid response to KdO_2_-Lipid A proceeds in three overlapping phases, highlighting how multiple layers of metabolism (concentrations, fluxes, and enzymes) coordinate to shape phase-specific adaptations. Figures E and F show flux and enzyme labels with the corresponding substrates in parentheses, where the same enzyme can metabolize multiple substrates. Since fluxes are completely resolved by the end of phase 3, the color coding of fluxes into resolving and non-resolving groups is based on the enzyme that catalyzes that reaction. Plots created with MATLAB.

Using the LIPID MAPS sphingolipid concentration data (supplemental Fig. S1) and our flux model algorithm, we generated predictions of metabolic fluxes (supplemental Fig. S2) and enzyme activities (supplemental Fig. S3). However, the number of these variables and their dynamic nature make global insights illusive. To gain a global overview of how sphingolipid metabolism evolves over the 24-h time course, we performed principal component analysis (PCA) on all three datasets—lipid concentrations, metabolic fluxes, and enzyme activities—extracted from KLA-stimulated RAW 264.7 macrophages. Despite measuring different levels of the pathway, all three PCA plots (Fig. 3A–C) revealed a similar trajectory that naturally separates into three phases: Phase 1 (0–4 h), Phase 2 (4–12 h), and Phase 3 (12–24 h). These phases are indicated by shifts in the first two principal components (PC1 and PC2), which together explain the majority of the variance in each dataset. The color scale in Figure 3 highlights time progression, and arrows denote the transition boundaries between phases.

Strikingly, each of the three PCA plots exhibits a smooth, coherent transition from early (Phase 1) to intermediate (Phase 2) and then to late (Phase 3) time points, suggesting that sphingolipid concentrations, fluxes, and enzymes are coordinately remodeled as inflammation proceeds. Notably, while the largest changes in PC scores occur between 4 and 12 h (transition into Phase 2), cells also appear to enter a distinct late-stage metabolic configuration after 12 h. This late shift underscores the possibility that certain lipids and enzymes remain persistently altered or begin to resolve only by the end of the experiment.

To identify which specific sphingolipid species, fluxes, or enzymes dominate each phase, we examined the loadings plots for PC1 and PC2 (Fig. 3D–F). Key metabolites such as ceramide, sphingosine, and their phosphorylated forms often show high loadings, indicating that they explain a substantial portion of the variance and help define major time-dependent transitions. Similarly, fluxes associated with sphingomyelin synthase (SMS) or ceramidase (CDase), and enzyme activities including sphingosine kinase (SK) and sphingomyelinase (SMase), cluster in ways that differentiate Phase 1 from Phase 2/Phase 3. In these figures, the notation “Enzyme (Substrate)” indicates that the enzyme is capable of using multiple substrates and the enzyme uses the indicated substrate for that reaction. These loading patterns point to distinct metabolic programs across early, intermediate, and late inflammatory responses—for example, ceramide production versus sphingosine phosphorylation—and reinforce the concept that macrophages rewire their sphingolipid metabolism in stage-specific patterns. Such phase-wise coordination implies that macrophages do not simply increase or decrease a single sphingolipid species; rather, they dynamically restructure the entire network in ways that likely underlie functionally distinct inflammatory and resolution states.

### Partial versus complete resolution of sphingolipid metabolism by Phase 3

**Fig. 4:**
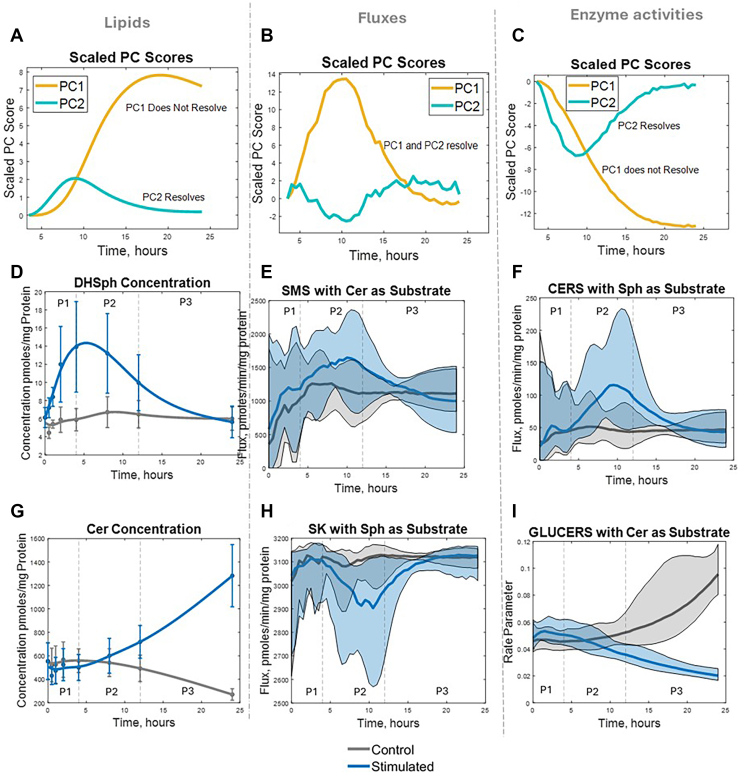
Time-course analyses reveal partial resolution of many sphingolipid species and enzyme activities—but complete resolution of fluxes—by Phase 3. A–C: Scaled principal component (PC) scores over time for sphingolipid concentrations (A), fluxes (B), and enzyme activities (C). PC2 largely returns to baseline by the end of Phase 3 (partial resolution) in the sphingolipid and enzyme datasets, whereas PC1 remains shifted. In contrast, both PC1 and PC2 are resolved in the flux dataset by the end of Phase 3. D–F: Representative examples of resolving behaviors for specific lipids (D), fluxes (E), and enzyme activities (F). G–I: Representative examples of non-resolving behaviors for specific lipids (G), fluxes (H), and enzyme activities (I). Each plot shows the time course under stimulated (blue) and control (gray) conditions, with the three phases indicated. “Resolving” variables return near control levels by the end of Phase 3, whereas “nonresolving” ones remain elevated or suppressed. Vertical dividers mark Phase 1 (0–4 h), Phase 2 (4–12 h), and Phase 3 (12–24 h). These examples illustrate how the overall PCA trajectories (A–C) are driven by diverse molecular outcomes at the level of individual lipids, fluxes, and enzymes. In (D–I), P1, P2, and P3 refer to phases 1, 2, and 3, respectively. In (E, F, H, and I), the colored bands around the mean contain the middle 68% of estimation results. Plots created with MATLAB.

To determine the extent of recovery in sphingolipid metabolism over the 24-h time course, we compared stimulated versus control cells in terms of (A) sphingolipid concentrations, (B) fluxes, and (C) enzyme activities using scaled principal component (PC) scores (Fig. 4A–C). In the sphingolipid and enzyme datasets (Fig. 4A, C), PC2 largely returns to near-control levels by 24 h, but PC1 remains markedly shifted. This suggests that, by the end of Phase 3, a subset of lipids and enzymes does revert toward pre-stimulation values (“resolving” components), whereas others remain altered (“nonresolving”) and could potentially drive sustained inflammatory or pro-survival signaling.

By contrast, both PC1 and PC2 in the flux dataset (Fig. 4B) return to baseline levels by 24 h. Thus, at the level of reaction rates, the macrophage sphingolipid network appears to restore its global flux patterns, even if certain metabolites and enzymes do not completely normalize. This mismatch between flux recovery and partial metabolite/enzyme recovery indicates that, after 12–24 h, cells maintain a stable flow of sphingolipid intermediates but with altered pool sizes or enzyme expression.

To illustrate these behaviors, Figure 4D–F presents representative time-course plots of “resolving” concentrations, fluxes, and enzyme activities while Figure 4G–I presents representative plots of “nonresolving” concentrations, fluxes, and enzyme activities. Vertical lines at 4 h and 12 h delineate the same three phases identified earlier—Phase 1 (0–4 h), Phase 2 (4–12 h), and Phase 3 (12–24 h). For instance, one sphingolipid class may return near the control level (Fig. 4D, resolving) while another remains elevated through 24 h (Fig. 4G, nonresolving). A similar dichotomy emerges in enzyme activities (Fig. 4F, I), showing how certain nodes in the pathway can reset by Phase 3, whereas others appear locked in an inflammatory or adaptive state. These findings underline the multi-layered complexity of macrophage sphingolipid regulation, where dynamic shifts in flux can compensate for persistent deviations in metabolites or enzyme levels to achieve a quasi-homeostatic state by the end of Phase 3. In Figure 4E, F, H, and I, the confidence intervals of the flux and enzyme activitiy predictions are shown as colored bands around the mean predictions.

### Validation of model predictions

**Fig. 5:**
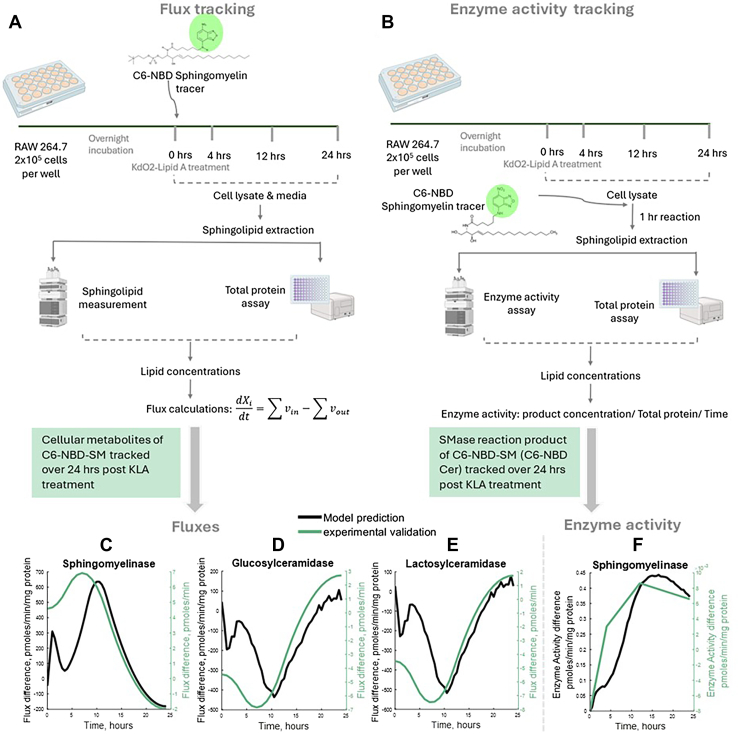
Experimental validation of model predictions. A: Experimental design for estimation of metabolic fluxes using C6-NBD-Sphingomyelin as a tracer. B: Experimental design of sphingomyelinase enzyme activity measurement using C6-NBD-Sphingomyelin as a substrate. C–E: Experimentally determined fluxes trace the same trajectory as the model prediction for (C) Sphingomyelinase, (D) Glucosylceramidase and Lactosylceramidase (E). Plots are shown as the difference in fluxes between unstimulated and KLA stimulated conditions. F: Experimentally determined enzyme activity of total sphingomyelinase traces the same trajectory as the model prediction. Plot is shown as the difference in activity between unstimulated and KLA stimulated conditions. Images and plots created with BioRender and MATLAB.

To validate the model’s predictions of fluxes and enzyme activities using orthoginal methods, we estimated metabolic fluxes using C6-NBD Sphingomyelin as tracer and measured the activity of sphingomyelinase (acid plus neutral) as a representative enzyme. Figure 5A, B show the experimental design for the two experiments. Figure 5C–E shows that the difference in the model’s predictions of flux between the control and KLA-treated conditions matches with that obtained experimentally using the NBD-sphingolipid tracer, for the fluxes through sphingomyelinase, glucosylceramidase, and lactosylceramidase. Similarly, the difference in total SMase activity observed using the NBD-substrate matches that of the original model predictions (Fig. 5F).

### Phase-Specific shifts in the Sphingolipid network reflect coordinated flux modulations

**Fig. 6:**
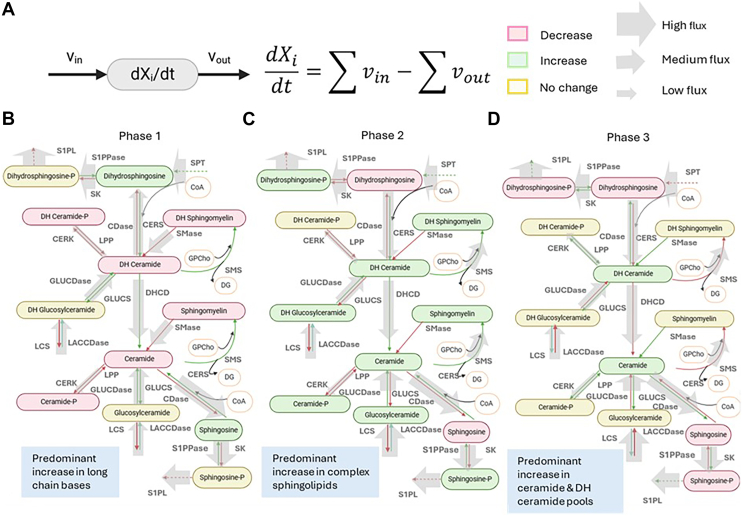
Coordinated flux changes drive shifts in sphingolipid concentrations over three phases of KdO_2_-Lipid A stimulation. A, Legend showing how features in network map relate to flux equations and variables. B–D: Network diagrams for (B) Phase 1, (C) Phase 2, and (D) Phase 3, depicting how lipid concentrations and fluxes change over time. Lipid boxes are color-coded as follows: green for increased concentration, red for decreased, and yellow for unchanged. Arrows are likewise colored to indicate increased (green), decreased (red), or unchanged (yellow) flux. Gray arrows denote net reaction directions for each reaction pair, with arrow thickness proportional to flux magnitude. In Phase 1, mass predominantly flows from complex sphingolipids (e.g., sphingomyelin, ceramide 1 phosphate, ceramide) toward long-chain bases (sphingosine, dihydrosphingosine). In Phase 2, complex sphingolipid pools are rebuilt and expanded, while accumulated long-chain bases are converted into their phosphorylated forms. Finally, in Phase 3, remaining long-chain bases and base phosphates are removed, concurrent with continued expansion of the ceramide pool. Images created with BioRender.

To better visualize how fluxes and concentrations co-evolve across the three phases, we mapped the observed changes onto the sphingolipid network for Phases 1, 2, and 3 (Fig. 6B–D). Consistent with the flux analyses, Phase 1 (0–4 h) is characterized by a net flow from complex sphingolipids (such as sphingomyelin, ceramide 1 phosphate, and ceramide) toward long-chain bases (sphingosine, dihydrosphingosine). In Figure 6B, this manifests as red arrows or boxes for several complex species, indicating decreases in their concentrations/fluxes, paired with green boxes/arrows for Sph and DHSph routes. This is further emphasized by the directionality of the gray arrows, which indicate the direction of the net flux between each forward/reverse enzyme pair. These findings suggest an early, rapid dismantling of certain “higher-order” sphingolipid pools, presumably to fuel signaling processes mediated by free bases.

By Phase 2 (4–12 h), the metabolic emphasis shifts. Many complex sphingolipids (eg, sphingomyelin, glucosylceramide) rebound or expand (Fig. 6C, green boxes), supported by increased flux through synthases (e.g., SMS, GLUCERS). Meanwhile, a portion of the accumulated long-chain bases is channeled into their phosphorylated forms (SphP, DHSphP). These phosphorylated bases also appear in green for flux or concentration in certain nodes, reflecting ongoing pro-survival or pro-migratory signaling. The data suggest that macrophages actively restore higher-order lipid pools while diversifying base intermediates via phosphorylation—possibly to balance pro-inflammatory and reparative processes.

In Phase 3 (12–24 h), most long-chain bases and their phosphorylated derivatives are downregulated (red boxes/arrows in Fig. 6D), implying a partial resolution of the early and intermediate accumulations. Many of the complex sphingolipids (e.g., sphingomyelin and glucosylceramide) stabilize at new elevated levels (yellow boxes). Notably, ceramide and dihydroceramide remain elevated or continue to expand (green), suggesting that these pools either are still actively synthesized or have not yet returned to baseline. This ongoing ceramide enrichment could underlie persistent pro-inflammatory signaling—or a preparation phase for further membrane remodeling—in late-stage inflammation. Overall, the phase-specific patterns provide a system-level view of how core sphingolipid pools ebb and flow through each stage of the macrophage response. Such coordinated flux modulations help explain how macrophages finely tune sphingolipid levels for diverse functional outcomes—ranging from cell signaling to membrane biogenesis—over the full inflammatory timeline.

### Key enzymatic fluxes drive major shifts in Sphingolipid concentrations

**Fig. 7:**
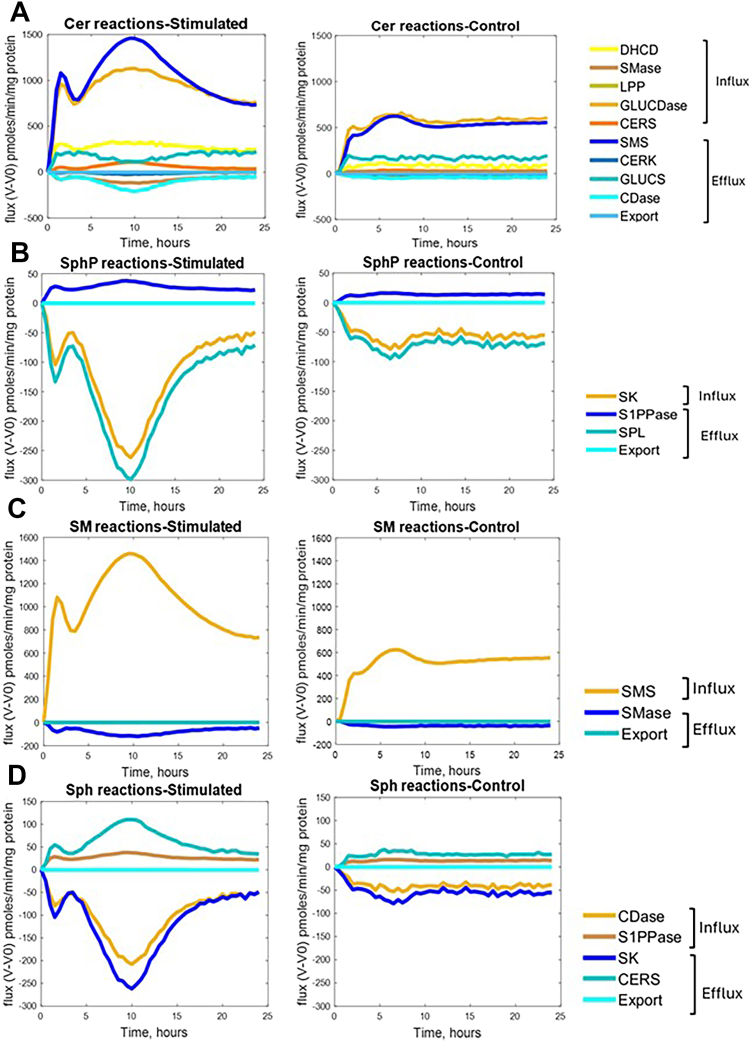
A few “key” fluxes dominate sphingolipid concentration changes after KdO_2_-Lipid A stimulation. A: ceramide (Cer) levels are primarily regulated by sphingomyelin synthase (SMS) and glucosylceramidase (GLUCDase) fluxes. B: Sphingosine phosphate (SphP) concentrations hinge on sphingosine kinase (SK) and sphingosine-1-phosphate lyase (SPL) activities. C: Sphingomyelin (SM) concentrations are largely determined by changes in SMS flux. D: Sphingosine (Sph) concentrations respond to SK and ceramidase (CDase) fluxes. In each panel, flux trajectories are shown as deviations from their baseline (t = 0) values. Notably, despite the broader complexity of sphingolipid metabolism, a small subset of enzymatic reactions appears to drive most of the observed concentration shifts in these four metabolites. This finding underscores the potential for targeted modulation of just a few fluxes—rather than tackling the entire pathway—to achieve substantial changes in sphingolipid homeostasis and macrophage inflammatory response. Plots created with MATLAB.

The complexity of sphingolipid metabolism results in multiple control strategies for the sphingolipid concentrations. For ceramide, which showed persistent elevation through Phase 3, control of concentration seems to be distributed across multiple fluxes: First, sphingomyelin synthase (SMS) and glucosylceramidase (GLUCDase) show by far the largest changes in flux in the stimulated group compared to the control group (Fig. 7A, B). Because GLUCDase produces ceramide and SMS consumes ceramide, the net effect is the flow of mass from GLUCER to SM. These two large fluxes usually balance each other out, but SMS dominates during Phase 2, resulting in a net loss of ceramide. At the same time, the other smaller fluxes, such as dihydroceramide desaturase (DHCD), contribute mass to the ceramide pool, resulting in a net increase in concentration. This suggests that ceramide accumulation during inflammation stems from a balance of the loss of mass during flow from glucosylceramide to sphingomyelin and the gain of mass from synthetic pathways. Notably, these results suggest that a small change in SMS or GluCDase would disrupt the balance between these two reactions and allow for significant changes in ceramide dynamics.

Sphingosine-1-phosphate (SphP) levels, critical for pro-survival signaling, are tightly controlled by the opposing actions of just 2 enzymes: sphingosine kinase (SK) and sphingosine-1-phosphate lyase (SPL) (Fig. 7B). The substantial drop in SPL flux during Phase 2 allows for significant accumulation of SphP, potentially moderating pro-survival signals as the inflammatory response progresses. This finding reveals a previously unappreciated temporal control point in sphingolipid-mediated inflammation.

Sphingomyelin concentrations track almost exclusively with SMS flux (Fig. 7C), highlighting this enzyme as a master regulator of membrane sphingolipid composition during inflammation. The dramatic increase in SMS flux during Phase 2 suggests that extensive membrane remodeling coincides with the intermediate inflammatory response. Meanwhile, sphingosine levels are primarily regulated by the coordinated activities of SK and ceramidase (Fig. 7D), with their fluxes showing strong temporal correlation but opposite directions during Phase 2. Similar patterns of concentrations being dominated by a few key fluxes emerged for other sphingolipids as well (supplemental Fig. S4). The dominant fluxes for all sphingolipids are summarized in Table 6.

**Table 6:** Dominant Fluxes for Each Sphingolipid

No.	Sphingolipid	Dominant Fluxes
1.	Dihydrosphingosine	SPT, CDase
2.	Dihydrosphingosine Phosphate	SPL, SK
3.	Dihydroceramide	GLUCDase, SMS
4.	Dihydrosphingomyelin	SMS
5.	Dihydroceramide Phosphate	CERK, LPPase
6.	Dihydroglucosylceramide	GLUCDase
7.	Ceramide	GLUCDase, SMS
8.	Sphingomyelin	SMS
9.	Ceramide Phosphate	CERK, LPP
10.	Glucosylceramide	LACCDase, GLUCDase
11.	Sphingosine	SK
12.	Sphingosine Phosphate	SPL

These findings reveal that while the sphingolipid network contains numerous interconnected reactions, therapeutic targeting of just a few key enzymes - particularly SMS, GluCDase, SK, or SPL - might be sufficient to reshape the inflammatory sphingolipid landscape. The temporal profiles of these dominant fluxes also suggest that the timing of such interventions could be crucial, with Phase 2 representing a particularly dynamic window for metabolic modulation.

### Sensitivity analysis identifies critical regulatory points in the Sphingolipid network

**Fig. 8:**
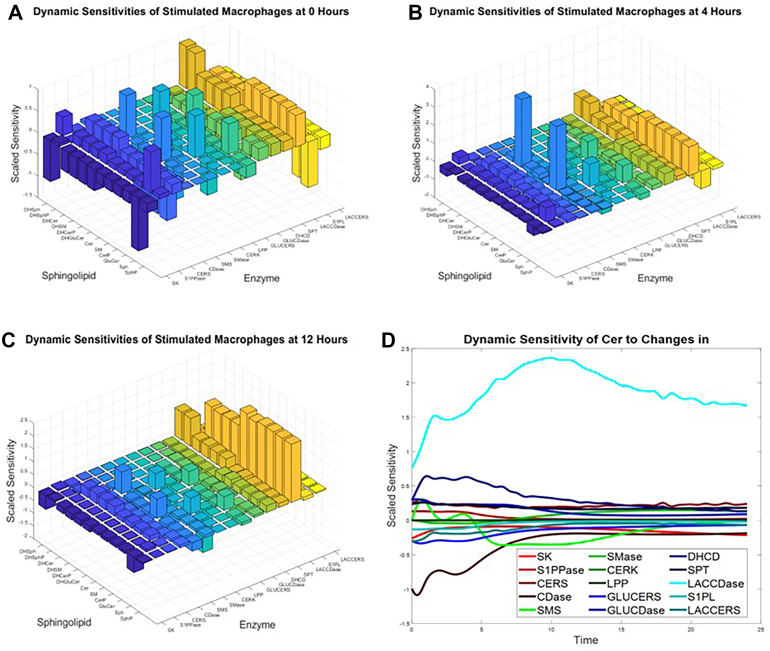
Sensitivities of Sphingolipids to Enzymes Change over Time, but a Small Number of enzymes Dominate. The scaled sensitivities of sphingolipid concentrations with respect to enzyme activities were calculated at (A) 0 h, (B) 4 h, and (C) 12 h post KLA stimulation. (D) Sensitivity of ceramide concentration to changes in enzyme activities throughout the entire 24-h experiment post KLA stimulation. Plots created with MATLAB.

**Supplementary Figure S5:**
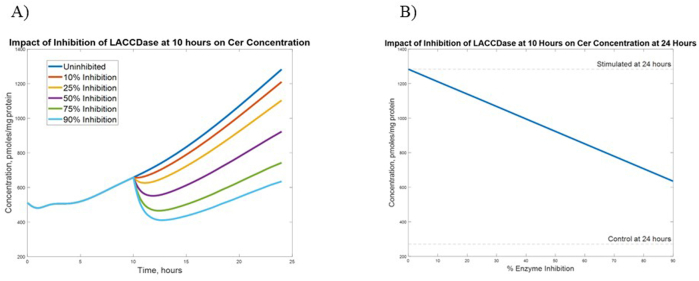


**Supplementary Figure S6:**
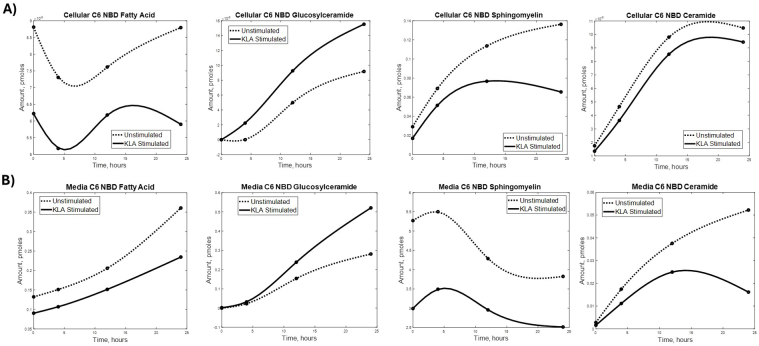


To understand how perturbations in enzyme activities influence sphingolipid levels, we performed dynamic sensitivity analysis. Based on this analysis, we produced sensitivity matrices mapping the sensitivities of lipid concentrations to changes in enzyme activities at 0, 4, and 12 h post-KLA stimulation. (Fig. 8A–C). The matrices reveal that at a given time point, each lipid responds strongly to only a few of the enzymes in the network. However, those influential lipids change as time progresses. This pattern suggests that targeting specific enzymes in specific time windows could achieve selective control over particular sphingolipid species. Ceramide sensitivity analysis illustrates this principle (Fig. 8B). Among the network's enzymes, LacCDase shows the highest sensitivity coefficient for ceramide at all time points, peaking at 10 h, while other enzymes like CDase and DHCD show modest sensitivities only in Phase 1 and SMS shows modest sensitivity only in Phase 2. This indicates that ceramide levels could be effectively modulated through LacCDase activity. This is consistent with the results from Figure 7, which identified GLUCDase as a key flux into the ceramide pool because changes in flux from LACCDase must flow through GLUCDase to reach ceramide. To test this prediction, we simulated progressive inhibition of LacCDase at 10 h, the time identified to have the highest sensitivity (supplemental Fig. S5). The model shows that 24 h ceramide concentrations decrease steadily with increasing LacCDase inhibition at 10 h, although the concentration is never able to completely reach the control level. This finding suggests that partial enzyme inhibition might be sufficient to reduce elevated ceramide levels observed during inflammation. The sensitivity matrix also reveals unexpected network relationships. For instance, dihydrosphingosine-based species show distinct sensitivity patterns compared to their sphingosine-based counterparts, suggesting separate regulatory mechanisms for these precursor pools. These insights from our computational analysis provide a quantitative framework for predicting how targeted enzymatic interventions might propagate through the sphingolipid network.

### Integration of sphingolipid dynamics with macrophage functional states

**Fig. 9:**
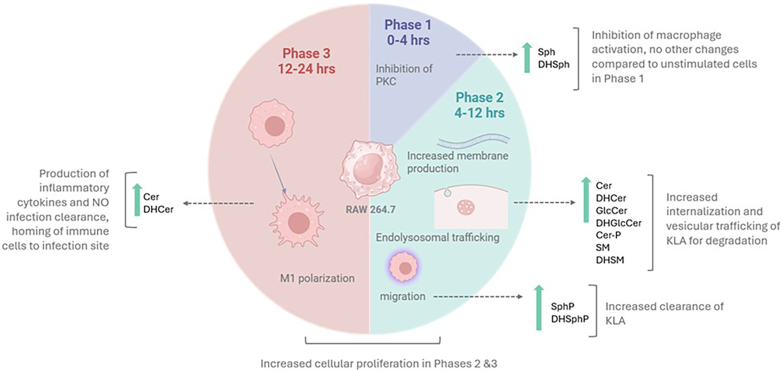
Three-phase macrophage response to KdO_2_-Lipid A involves dynamic lipid remodeling and functional adaptations. Phase 1 (0–4 h) features relatively limited changes in macrophage activation alongside the inhibition of PKC, with cells remaining close to baseline in both morphology and sphingolipid levels. Phase 2 (4–12 h) is characterized by increased membrane production and pronounced endolysosomal trafficking, accompanied by upregulated sphingolipid species (e.g., Cer, DHCer, GlcCer, and others). This phase supports enhanced internalization and vesicular processing of KLA for downstream immune signaling. Phase 3 (12–24 h) culminates in robust M1 polarization, with elevated Cer and DHCer contributing to the production of inflammatory cytokines, nitric oxide (NO), and the recruitment of additional immune cells. Meta-analysis of the literature and our data indicates that each phase integrates distinct metabolic programs to drive effective responses against inflammatory stimuli. The sequential upregulation of sphingolipids during Phases 2 and 3 supports increased cellular proliferation, membrane remodeling, and ultimately, inflammatory effector functions essential for infection clearance. Images created with BioRender.

Our temporal analysis of sphingolipid metabolism reveals distinct metabolic programs that align with key functional transitions in macrophage activation (Fig. 9). In Phase 1 (0–4 h), the selective increase in sphingosine and dihydrosphingosine, known inhibitors of protein kinase C (PKC), suggests an initial regulatory checkpoint that temporarily constrains full inflammatory activation (1). This early phase shows minimal changes in other sphingolipid species, indicating that cells maintain near-baseline membrane composition while establishing appropriate activation thresholds. Phase 2 (4–12 h) marks a dramatic metabolic shift characterized by enhanced membrane production and vesicular trafficking. The coordinated upregulation of complex sphingolipids, including ceramide, dihydroceramide, and glucosylceramide, supports increased endolysosomal activity (6).

This metabolic remodeling also coincides with elevated S1P and dhS1P levels, which promote cell survival and migration (13). The LIPID MAPS dataset reveals increased DNA content during this phase, suggesting that sphingolipid changes also support heightened cellular proliferation (20). By Phase 3 (12–24 h), the sphingolipid landscape shifts toward an inflammatory profile dominated by ceramide and dihydroceramide accumulation. This metabolic signature aligns with M1-like polarization (63), enabling enhanced production of inflammatory mediators and recruitment of additional immune cells. The sustained elevation of these pro-inflammatory sphingolipid species, even as other metabolites begin returning to baseline, suggests their importance in maintaining appropriate immune activation (15).This three-phase model demonstrates how macrophages orchestrate sequential sphingolipid programs to achieve effective inflammatory responses while maintaining essential cellular functions. The temporal coordination between lipid remodeling and functional adaptations provides new insight into the metabolic regulation of innate immunity (6).

## Discussion

Our systematic analysis of sphingolipid metabolism in activated macrophages reveals a coordinated three-phase response that provides new mechanistic insights into inflammatory regulation. The temporal evolution from early sphingolipid mobilization to later partial resolution demonstrates how macrophages orchestrate metabolic programs to support distinct functional states.

Phase 1 (0–4 h) is marked by an increase in sphingosine and dihydrosphingosine, two long-chain bases that can inhibit protein kinase C and thus transiently dampen TLR4-mediated signaling (1, 11, 64). This early checkpoint could serve as a regulatory buffer before full-scale macrophage activation. By Phase 2 (4–12 h), we observe pronounced accumulations of complex sphingolipids (e.g., sphingomyelin, glucosylceramide) and the bioactive lipids S1P and DHS1P, potentially linked to membrane biogenesis and endolysosomal trafficking (20, 65). Enhanced S1P levels in particular are associated with cell survival, migratory signaling, and anti-apoptotic pathways (15, 66). Finally, Phase 3 (12–24 h) is typified by ceramide and dihydroceramide accumulation, reminiscent of an M1-like pro-inflammatory phenotype (63). Although many metabolites and fluxes partially return toward baseline, ceramide elevations often persist, suggesting incomplete resolution that could maintain a heightened inflammatory tone if not further regulated (7, 11).

One emerging concept is that macrophages can retain a “metabolic memory” of prior inflammatory signals, influencing how they respond to subsequent challenges (6). Our data illustrate that certain sphingolipid pools (e.g., ceramide) and enzymes remain altered into Phase 3, possibly priming macrophages for heightened responses if stimulation recurs. Intriguingly, anti-inflammatory cytokines such as IL-10 have been shown to constrain sphingolipid metabolism and reduce excessive inflammatory signaling (64, 65). It is plausible that IL-10 or related signals might tip the balance of late-phase sphingolipid metabolism back toward S1P or ceramide-1-phosphate, fostering a more resolving or reparative macrophage state (8, 66). Future experiments manipulating IL-10 levels or downstream effectors in conjunction with time-resolved sphingolipid profiling could clarify how these regulatory axes influence “metabolic memory” within the macrophage.

Our sensitivity analysis suggests that targeting key enzymes (e.g., lactosylceramidase, sphingomyelin synthase) at different phases can exert outsized impacts on sphingolipid concentrations. This aligns with recent findings that sphingolipid metabolism is a druggable pathway for modulating inflammation (16, 67). Importantly, the timing of such interventions appears crucial. In Phase 2, for example, the surge in S1P and other phosphorylated bases may present an optimal window to either attenuate pro-survival signals or enhance resolving mechanisms—depending on the clinical context (68, 69). Conversely, inhibiting ceramide synthesis or conversion in Phase 3 could help prevent chronic inflammation and tissue damage. By providing a quantitative and temporal perspective, our approach opens the door to designing phase-aligned therapies where enzyme inhibition or activation is timed to the metabolic transitions most likely to influence outcome. Similar methods were used by Alvarez-Vasquez to create a biochemical systems theoretical model of sphingolipid metabolism in Saccharomyces cerevisiae to predict the effects of flux modulations, genetic enzyme perturbations and the impact of inositol regulation with high agreement to experimental results. Importantly, the model also allowed the simulation of changes in fatty-acid precursors that are difficult or test experimentally, thereby demonstrating its utility in in-silico “thought experiments” (70). Recent studies have highlighted the complexity of immunometabolism, with macrophages integrating glycolysis, fatty acid oxidation, arginine metabolism, and sphingolipid pathways to fine-tune pro- versus anti-inflammatory phenotypes (1, 15). Our observation that S1P and ceramide fluxes strongly shape cell fate decisions underscores the synergy between lipid remodeling and classical immune signaling. Moreover, the partial resolution we observed after 24 h may reflect macrophages striving for a balanced inflammatory response—strong enough to neutralize pathogens yet not so excessive as to induce bystander tissue damage (6, 66). Understanding how these sphingolipid programs intersect with other metabolic circuits, including the TCA cycle or pentose phosphate pathway, represents a significant next step (16).

### Limitations

Despite offering new insights, our study has certain limitations. First, enzyme activities were inferred from literature sources rather than measured directly in RAW 264.7 macrophages; thus, specific reaction rates may differ in vivo or under varied culture conditions (10). Second, our bulk cell analysis cannot capture potential subcellular compartmentalization of sphingolipid metabolism or cell-to-cell heterogeneity. Recent advances in single-cell lipidomics using trapped ion mobility separation (71) and spatial mapping techniques (14, 21) provide valuable insights into the spatial distribution and heterogeneity of sphingolipid dynamics during macrophage activation, and explore how different macrophage subpopulations might exhibit distinct metabolic programs during inflammation (72). Third, macrophages in tissues encounter multicellular contexts, gradients of cytokines, and dynamic oxygen levels—all absent in simplified in vitro systems. Future work could examine how local sphingolipid gradients (e.g., in fibrotic lung, atherosclerotic plaques, or traumatic tissue loss) shape macrophage responses in vivo (3). Fourth, although we explored a range of flux behaviors, additional multi-omics analyses (e.g., transcriptomics, proteomics) might further elucidate how sphingolipid metabolism integrates with other immunometabolic processes. Nonetheless, our model provides a valuable starting point for predictive analyses, bridging in vitro measurements with potential in vivo applications (16, 69).

## Conclusion

Together, these findings establish a quantitative foundation for understanding how macrophages coordinate sphingolipid metabolism during inflammation. By delineating a three-phase progression—early dampening (Phase 1), complex sphingolipid/S1P buildup (Phase 2), and partial resolution with persistent ceramide (Phase 3)—we highlight temporal niches in which therapeutic interventions could be most effective. The identification of phase-specific control points (e.g., glucosylceramidase, sphingosine kinase) and metabolite “memory” (e.g., sustained ceramide elevations) has direct implications for designing more targeted anti-inflammatory therapies. Moving forward, integrating these insights with complementary immunometabolic data and in vivo validation may yield robust strategies to modulate macrophage-driven inflammation without broadly inhibiting their essential immune functions.

## Data availability

Time-series data used for modeling can be found at (9). Enzyme activities used for modeling can be found in the references listed in Table 4. All other data can be provided upon request to Edward A. Botchwey (Georgia Institute of Technology and Emory University, email: edward.botchwey@bme.gatech.edu).

## Supplemental data

This article contains supplemental data.

## Conflict of interest

The authors declare that they do not have any conflicts of interest with the content of this article.

## Figures and Tables

**Supplementary Figure S2-1:**
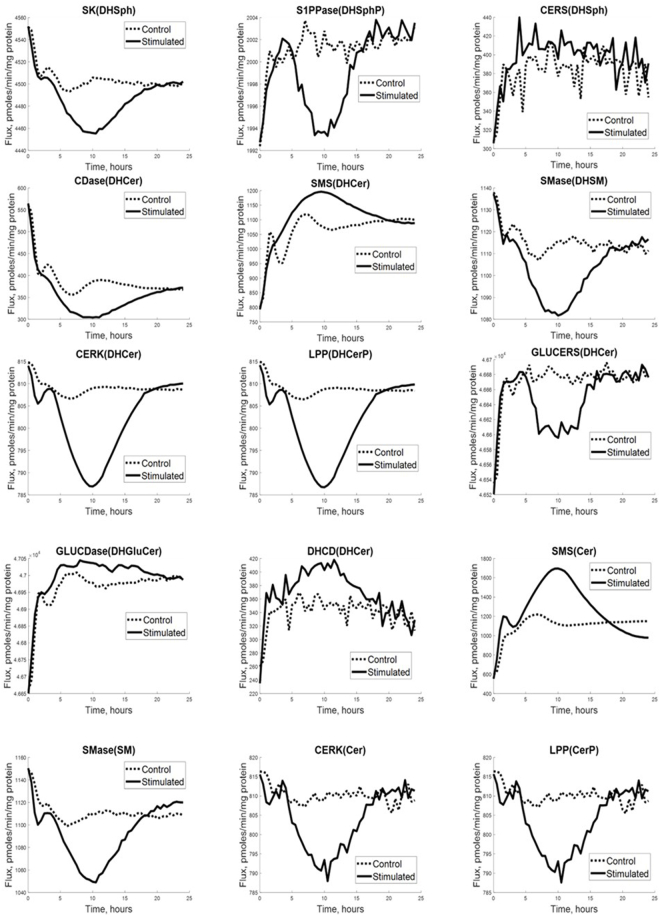
Fluxes Were Estimated Using Experimental Concentration Data and Dynamic Flux Estimation. Fluxes were estimated for control macrophages (dotted line) and stimulated macrophages (solid line) by using the derivatives of the concentration curves to solve a flux balance equation at each time point. The notion “Enzyme (Substrate)” in figure titles indicates that the enzyme is capable of using multiple substrates and for figure shown, the enzyme is using that specific substrate.

**Supplementary Figure S2-2:**
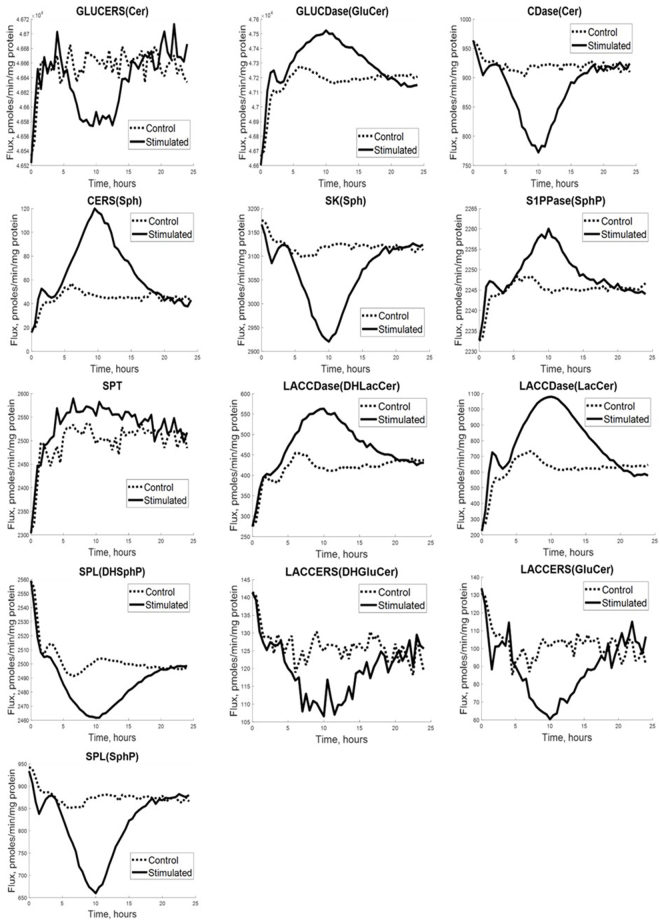


**Supplementary Figure S2-3:**
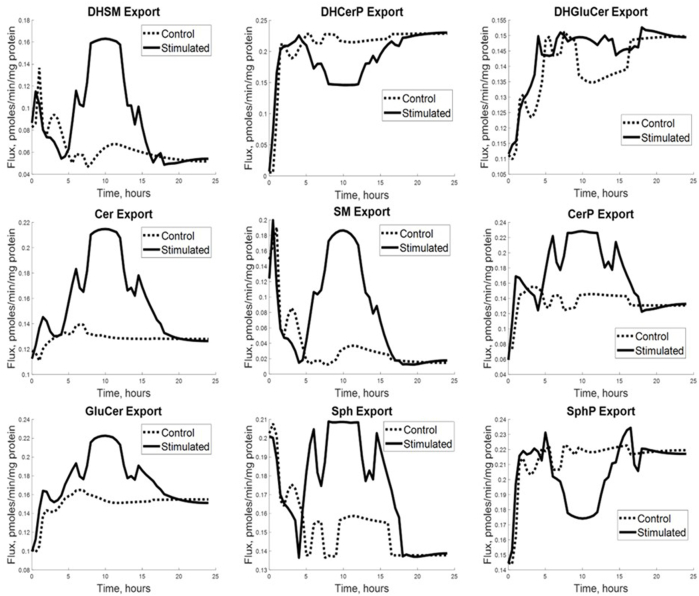


**Supplementary Figure S3-1:**
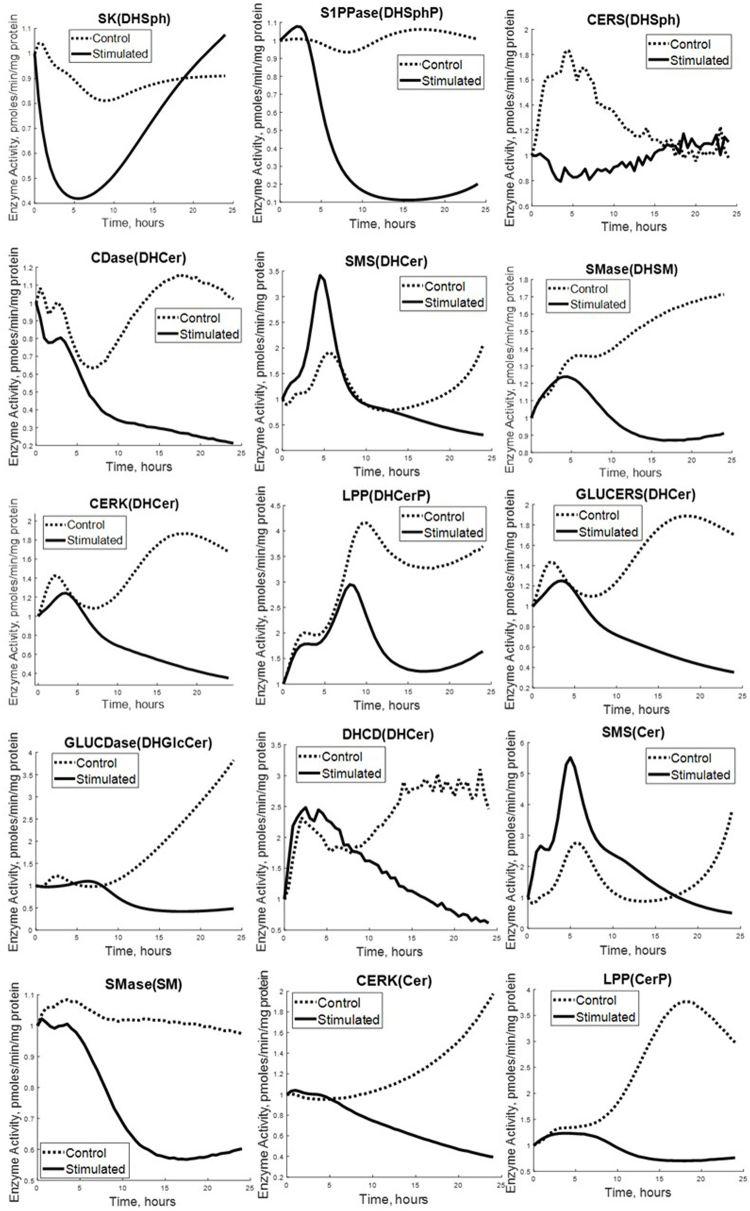
Enzyme Activities Were Estimated Using Experimental Concentration Data and Dynamic Flux Estimation. Enzyme activities were estimated for control macrophages (dotted line) and stimulated macrophages (solid line) by using the measured substrate concentrations, estimated flux values and kinetic rate equations.

**Supplementary Figure S3-2:**
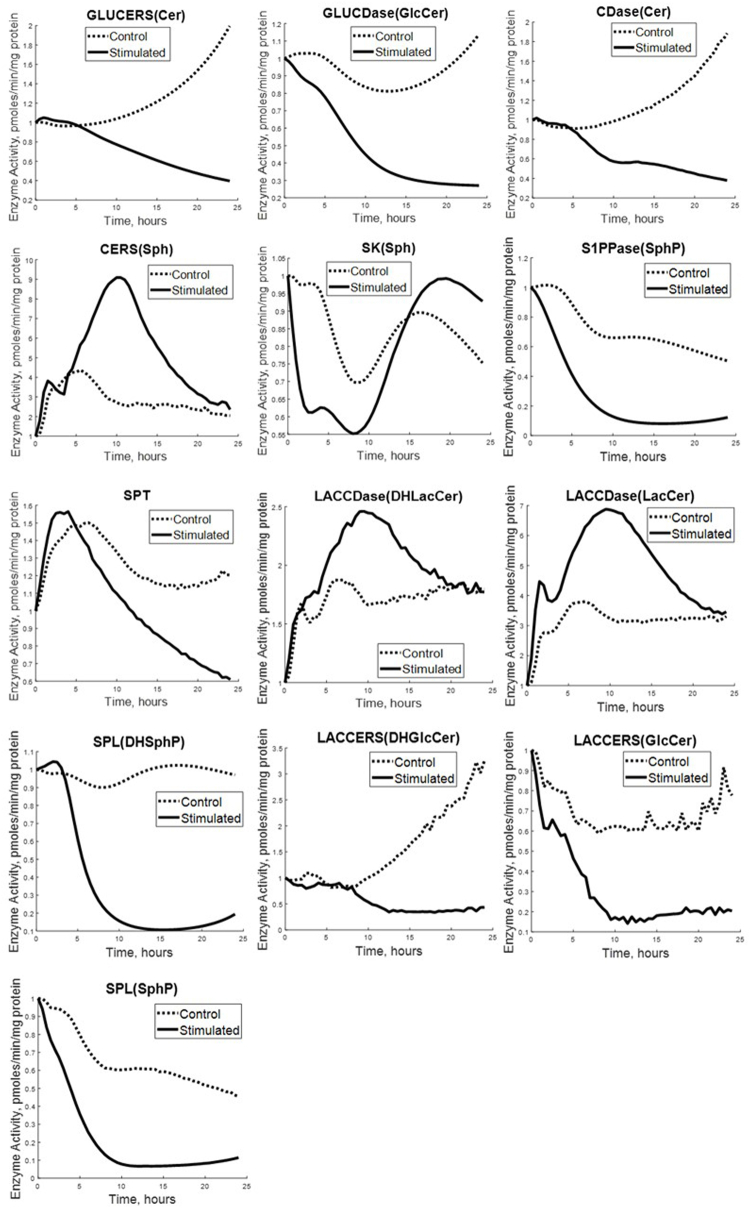


**Supplementary Figure S3-3:**
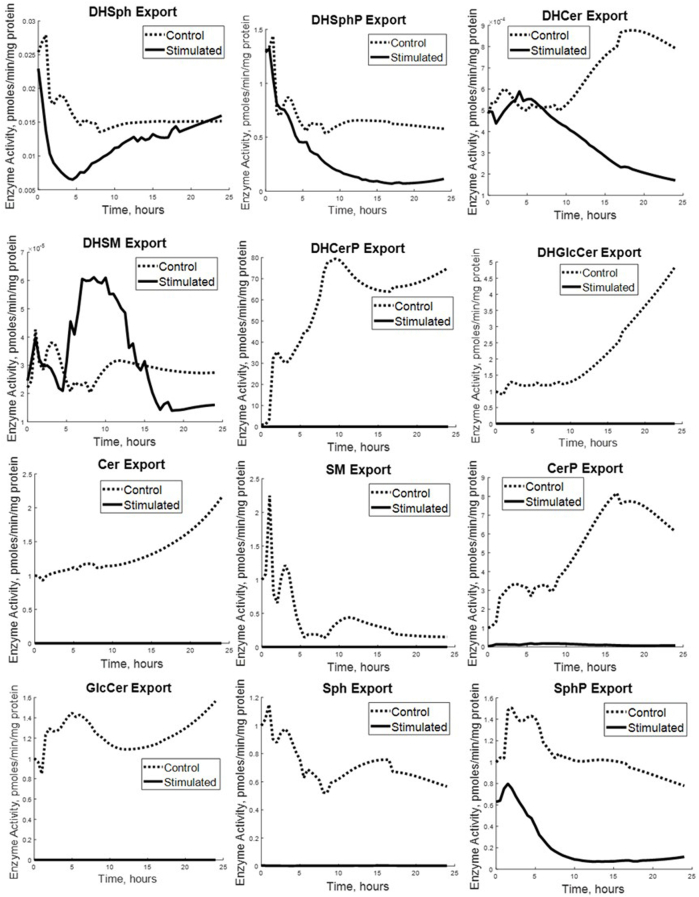


**Supplementary Figure S4-1:**
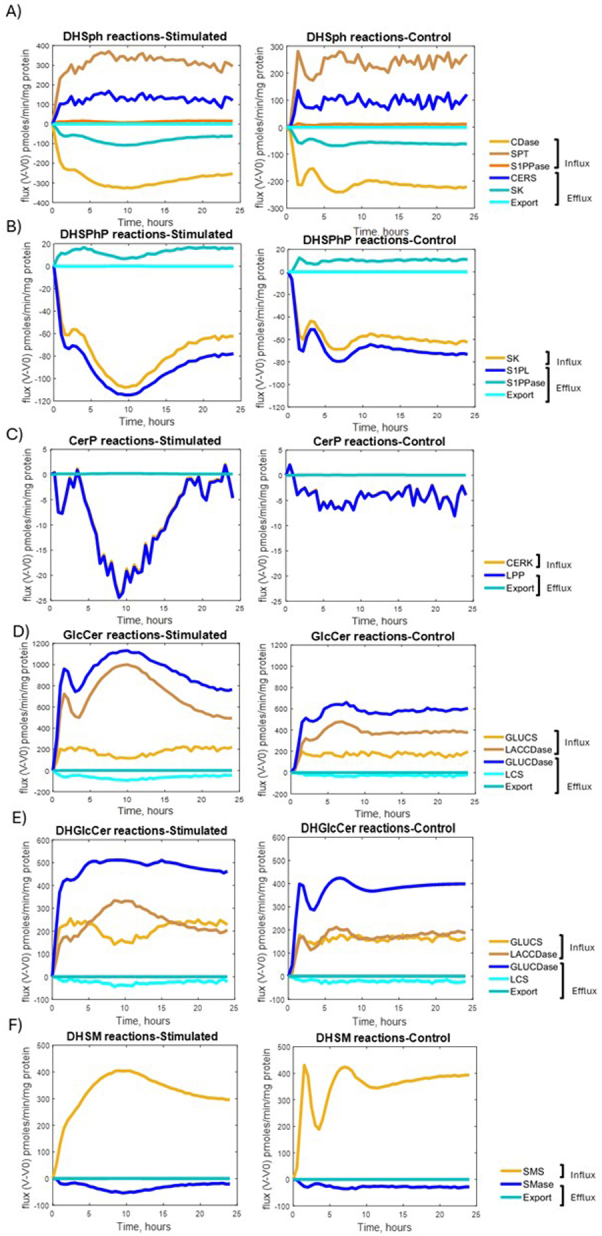
A Few “Key” Fluxes Dominate Sphingolipid Concentration Changes After KdO₂-Lipid A Stimulation. (A) Dihydrosphingosine (DHSph) levels are primarily regulated by serine palmitoyltransferase (SPT) and ceramidase (CDase) fluxes. (B) Dihydrosphingosine 1 phosphate (DHSphP) concentrations hinge on sphingosine kinase (SK) and sphingosine phosphate lyase (SPL) fluxes. (C) Ceramide 1 Phosphate (CerP) concentrations are largely determined by changes in ceramide kinase (CERK) and Lipid Phosphate Phosphatase (LPPase) fluxes. (D) Glucosylceramide (GluCer) concentrations respond to glucosylceramidase (GLUCDase) and lactosylceramidase (LACCDase) fluxes. (E) Dihydroglucosylceramide (DHGluCer) concentrations are largely determined by changes in GLUCDase flux. (F) Dihydrosphingomyelin (DHSM) concentrations respond to SMS flux. (G) Dihydroceramide (DHCer) concentrations are largely determined by changes in SMS and GLUCDase fluxes. (H) Dihydroceramide 1 Phosphate (DHCerP) concentrations respond to CERK and LPPase fluxes. In each panel, flux trajectories are shown as deviations from their baseline (t = 0) values. Notably, despite the broader complexity of sphingolipid metabolism, a small subset of enzymatic reactions appears to drive most of the observed concentration shifts in these eight metabolites. This finding underscores the potential for targeted modulation of just a few fluxes—rather than tackling the entire pathway—to achieve substantial changes in sphingolipid homeostasis and macrophage inflammatory response.

**Supplementary Figure S4-2:**
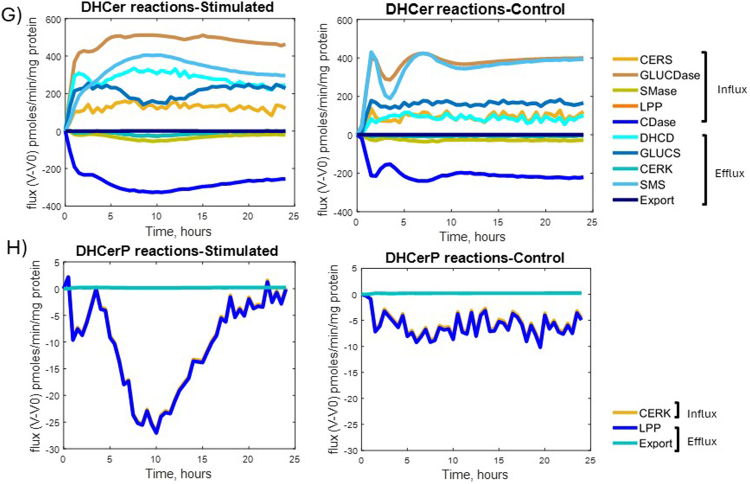


## Supplementary data

MacConcPlots_JLR

MacFluxPlots_JLR

MacNBDSMFluxModel_JLR

MackPlots_JLR
